# Recombinant Heparin—New Opportunities

**DOI:** 10.3389/fmed.2018.00341

**Published:** 2018-12-04

**Authors:** Charles Alexander Glass

**Affiliations:** TEGA Therapeutics, Inc., La Jolla, CA, United States

**Keywords:** anticoagulant heparin, heparan sulfate composition, recombinant heparin, growth factor binding, polysaccharide sulfation

## Abstract

Heparin and heparan sulfate (HS) are polydisperse mixtures of polysaccharide chains between 5 and 50 kDa. Sulfate modifications to discreet regions along the chains form protein binding sites involved in cell signaling cascades and other important cellular physiological and pathophysiological functions. Specific protein affinities of the chains vary among different tissues and are determined by the arrangements of sulfated residues in discreet regions along the chains which in turn appear to be determined by the expression levels of particular enzymes in the biosynthetic pathway. Although not all the rules governing synthesis and modification are known, analytical procedures have been developed to determine composition, and all of the biosynthetic enzymes have been identified and cloned. Thus, through cell engineering, it is now possible to direct cellular synthesis of heparin and HS to particular compositions and therefore particular functional characteristics. For example, directing heparin producing cells to reduce the level of a particular type of polysaccharide modification may reduce the risk of heparin induced thrombocytopenia (HIT) without reducing the potency of anticoagulation. Similarly, HS has been linked to several biological areas including wound healing, cancer and lipid metabolism among others. Presumably, these roles involve specific HS compositions that could be produced by engineering cells. Providing HS reagents with a range of identified compositions should help accelerate this research and lead to new clinical applications for specific HS compositions. Here I review progress in engineering CHO cells to produce heparin and HS with compositions directed to improved properties and advancing medical research.

## Cellular Production; An Attractive Source of Heparin/HS

Heparin and heparan sulfate (HS) are related polysaccharide chains with expression and structural similarities that give them similar properties but with some fundamental differences. Heparin and HS polysaccharide chains have backbones composed of the same repeating disaccharides that are, in both cases, modified by sulfation at various positions of the sugar residues. Biosynthesis of heparin and HS—initiation, elongation, and modification of the polysaccharide chains—proceed through the same enzymatic pathway in the Golgi (1, 2). Whereas, HS is displayed on the surfaces of all cells, heparin is exclusively stored in the cytoplasmic granules of mast cells. The functional characteristics of heparin and HS are determined by the sulfation patterns in localized regions of the polysaccharide chains. In HS, particular sulfation patterns in these localized regions form specific protein binding sites that serve as coreceptors for growth factors and cytokines (3).

HS sulfation patterns change during development and vary among different tissues, differentially regulating the effects of growth factor and cytokine signal transduction. These properties give HS important roles in a number of physiological and pathophysiological areas. *In vivo* heparin function is much less well-understood. Heparin may aid in the packaging and storage of histamine and other inflammatory mediators stored in mast cell granules that are released upon IgE stimulated degranulation. Structurally, heparin can be thought of as over-sulfated HS as it tends to be considerably more highly sulfated; including the high frequency of a characteristic trisulfated disaccharide, and the presence of the antithrombin 3 (AT3) binding site (see below) that is almost completely absent in HS. This structure is responsible for the potent anticoagulant properties for which pharmaceutical heparin is known and widely prescribed. Pharmaceutical heparin is a highly purified fraction of material prepared from animal tissue, largely porcine intestine, and formulated for intravenous or subcutaneous administration. *in vivo* heparin is sequestered in granules where it is not thought to have effects on blood clotting, although, on release, there may be protective effects on tissues from inflammatory cell invasion (4, 5). Like HS, heparin chains harbor numerous protein binding sites, although at a higher density due to the high levels of sulfation, and many non-anticoagulant physiological properties of heparin were identified retrospectively from patients treated with heparin to prevent blood clotting. Clinicaltrials.gov lists hundreds of clinical trials where patients treated with heparin were monitored for non-anticoagulant benefits, for example in sepsis and oncology.

Pharmaceutical heparin is a widely prescribed anticoagulant drug, with 300,000 doses administered daily in the U.S. and a worldwide market of over $7B (6–8). US regulatory agencies are concerned about the heparin supply because ~80% of the worldwide heparin API is produced from pig intestines in China. Heparin manufacturing is hard to regulate in China as evidenced by the heparin adulteration crisis in 2008 that led to allergic reactions and over 250 deaths worldwide. There is also concern as to whether the pig population can keep up with the growing global demand for heparin. To shore up the heparin supply chain, the FDA recently decided to consider reintroducing bovine heparin as an alternative source. Bovine heparin was allowed in the US until the 1990s when it was discontinued over concerns about bovine spongiform encephalopathy (BSE), aka mad cow disease (9). Mad cow disease was never detected in the US and has all but been eliminated in Europe (From the CDC in 2017: A rough estimate of this risk for the UK in the recent past, for example, was about 1 case per 10 billion servings) however, bovine heparin, as an animal-derived product, is still subject to contamination from animal tissues and shortages due to diseases in the animal population. Additionally, bovine heparin has different anticoagulant properties and would be dosed differently than porcine heparin (10–12). This complication is underscored by the Brazilian Pharmacopeia, which has developed two separate monographs for bovine and porcine intestinal heparin in anticipation of reintroduction of bovine heparin (10). Along with those concerns, heparin standardization only reflects anticoagulant activity, which depends on the antithrombin binding pentasaccharide. Other biological activities caused by non-anticoagulant polysaccharide sequences may differ between animal species leading to unexpected side effects (13). Cellular production of heparin offers an alternative that allows the entire supply chain to be under GMP control. Cellular production can be scaled to meet demands, and it would be much simpler and quicker to clean and restart a contaminated bioreactor than to restart a diseased animal population.

New oral anticoagulants (NOA) are gaining acceptance and market share because of more convenient routes of administration, decreased monitoring requirements and in some cases better safety profiles. How extensive this market infiltration becomes remains to be seen. Heparin is fast acting, completely reversible and currently has the advantage of efficacy and safety data from dosing patients globally for over 75 years. There are also clinical situations where heparin may be preferable, such as for patients with prosthetic heart valves as NOA have greater risks of valve thrombosis (14, 15), pregnancy where there is a lack of clinical experience (16–18), renal impairment as NOAs are renally excreted (19), severe liver diseases as NOAs are hepatically metabolized (19), and gastrointestinal disease as direct Factor Xa inhibitors have greater risks of intestinal bleeding (19).

Perhaps the real power of cellular production is the prospect of altering the structure of heparin, in order to improve its properties, or to produce polysaccharide chains that are tailored to the non-anticoagulant applications identified retrospectively. For example, in cardiopulmonary bypass surgery where heparin is the drug of choice (20), risks of heparin induced thrombocytopenia (HIT) associated with the high doses of heparin administered before and during surgery have already led to the introduction of alternative anticoagulant therapies. Recombinant heparin provides an opportunity to reduce the risk of HIT by engineering the molecular structure of cell-produced heparin.

Biotechnology has come a long way in eliminating the need to source medicines from animals. Heparin has been an exception, perhaps due to the large number of slaughtered pigs in China and the low (but rising) cost of labor there, but more importantly, producing recombinant heparin entails a higher level of complexity than producing a recombinant protein. Unlike recombinant proteins that are expressed by a single gene, heparin is synthesized in a complex metabolic pathway involving over 20 enzymes. Heparin polysaccharides are uniquely produced in mast cells but because mast cells are particularly difficult to propagate and maintain, they are not suitable for commercial production. The CHO cell line is an industry standard for producing recombinant therapeutic products. This familiarity may be an advantage from a regulatory standpoint, but another advantage is that CHO cells make relatively large amounts of HS. Producing recombinant heparin from CHO cells entails engineering the CHO cells genetically, to produce HS with the anticoagulant properties of heparin. Further genetic engineering could be aimed at reducing other protein binding substructures, for example, platelet factor 4 (PF-4) binding to reduce the risk of heparin-induced thrombocytopenia (HIT).

## Recombinant Heparin

**Figure 1:**
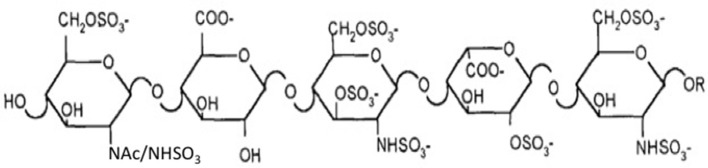
AT3 binding pentasaccharide.

Two major challenges confront the prospect of developing cell lines that produce recombinant heparin. The first challenge is the difference in composition between heparin and HS. Heparin inhibits coagulation by binding to AT3 and enhancing its activity (~1,000-fold). AT3 is a serine protease inhibitor that inhibits blood clotting by neutralizing thrombin (Factor IIa), Factor Xa and other serine proteases in the coagulation pathway. Heparin binds to AT3 via pentasaccharide binding motifs located along the heparin chains (see Figure 1). The AT3 binding pentasaccharide is present in about one third of heparin chains, while it is almost absent in HS (5) (see Table 1). Strong electrostatic interactions due to the high negative charge density in longer heparin sequences are also important for binding (21, 22). Thus, CHO cells must be engineered to produce highly sulfated polysaccharide chains containing sufficient AT3 pentasaccharide binding sites to meet the heparin standards for Factor Xa and Factor IIa assays.

**Table 1:** Properties of HS and heparin (22).

**Property**	**HS**	**Heparin**
Sulfates/hexosamine	0.6–1.8	1.8–2.4
GlcN *N*-sulfation	30–60%	≥80%
IduA content	20–50%	≥80%
Major disaccharide species	Variable	Trisulfated
Solubility in 2M KAc pH 5.7	+	–
Chains supporting AT3 binding	0–0.3%	~30%

**Figure 2:**
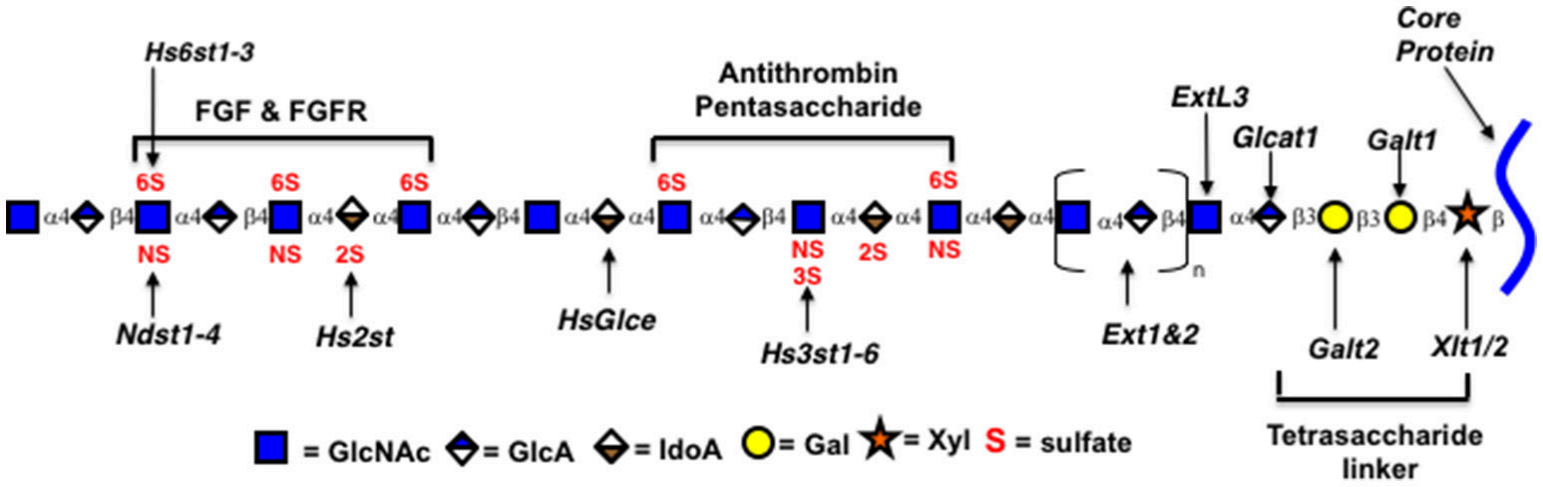
Genes encoding Heparin/HS biosynthetic enzymes. Xytl, xylosyltransferase; Galt, galactosyltransferase; Ext, exostosins, GlcNAc and GlcA transferases; Ndst1-4, GlcNAc *N*-deacetylase/*N*-sulfotransferase; Hs2st, uronyl 2-*O*-sulfotransferase; Hs3st1-6, glucosaminyl 3-*O*-sulfotransferase; Hs6st1-3, glucosaminyl 6-*O*-sulfotransferase; HsGlce, uronyl C5 epimerase; ExtL3, Exostosin Like Glycosyltransferase 3; Glcat1, glucuronyltransferase 1. Enzymatic isoforms indicated by numbers. Oligosaccharides identified by brackets.

Heparin and HS are members of a class of polysaccharides referred to as glycosaminoglycans (GAGs). The heparin/HS backbone is a polysaccharide synthesized as a copolymer of alternating D-glucuronic acid (GlcA) and *N*-acetyl-D-glucosamine (GlcNAc) residues by two enzymes, GlcNAc and GlcA transferases (Ext1 and Ext2; See Figure 2) (2). The polysaccharide chain is synthesized while attached to a core protein to form proteoglycan structures through a tetrasaccharide linker (GlcA-Gal-Gal-Xyl-). Specific protein binding sites are formed by modifications to short oligosaccharide regions along the backbone (22). Proteins bind through certain geometries of electrostatic charges on heparin formed by the modification enzymes that epimerize glucuronic acid to iduronic acid (glucuronic C-5 epimerase, Glce) and transfer negatively charged sulfate groups to the 2-*O*-position of iduronic acid (Hs2st), to the *N*-position of *N*-acetylglucosamine (four isozymes NDST1-4), to the 3-*O*-position of *N*-sulfoglucosamine (seven Hs3st isozymes), and to the 6-*O*-position of *N*-acetyl/sulfoglucosamine (three isozymes Hs6st1-3) (2). Research has provided clues to how these enzymes act on the polysaccharide during synthesis (1, 2, 5, 23).

- First, NDST deacetylates and sulfates a subset of GlcNAc residues to form GlcNS.
- Further *N*-deacetylation and sulfation of GlcNAc is preferred in regions proximal to GlcNS.
- The epimerase requires an upstream (toward the non-reducing end) GlcNS.
- The epimerase is inhibited by downstream (toward the reducing end) 6-*O*-sulfation.
- 6-*O*-sulfation generally requires prior *N*-sulfation or an adjacent GlcNS unit.
- 3-*O*-sulfation occurs in areas of high sulfate and iduronic acid.
- Sulfation compensation tends to maintain the wildtype net charge e.g., by increasing *N*- and 6-*O*-sulfation in the absence of 2-*O*-sulfation.

Sulfation levels are determined by the expression levels of the modification enzymes but how that is regulated is not well-known. Synthesis is not template driven, and none of the reactions in the biosynthetic pathway proceed to completion so there is structural heterogeneity among the polysaccharide chains in both size and structure. Typically, heparin and HS samples are characterized by compositional analyses, which quantify the disaccharides that make up the polysaccharide chains by their modifications. Table 1 compares structural and functional characteristics of heparin and HS.

The enzymes immediately relevant to increasing the number of AT3 binding sites can be predicted from the AT3 binding site structure (Figures 1, 2) and the contribution of each sulfate group to the AT3-pentasaccharide binding energy. The high affinity pentasaccharide terminates with a trisulfated disaccharide (residues 4 and 5 in Figure 1) (24). This is the most abundant disaccharide in heparin and it is relatively rare in HS. The AT3-pentasaccharide also contains a critical 3-*O*-sulfated group on the central glucosamine (residue 3) and a critical 6-*O*-sulfate group on the non-reducing end glucosamine (residue 1). These two sulfate groups are responsible for more than half of the binding energy of the interaction (25). 3-*O*-sulfate levels vary in HS from different cell types and tissues but are generally rare (5). While 2-*O*-sulfate is present in the canonical AT3-pentasaccharide sequence, it has been shown to be dispensable for high affinity binding to AT3 (23). These studies indicate that *N*-sulfate, 6-*O*-sulfate and 3-*O*-sulfate are critical components for high affinity binding. Disaccharide compositional comparisons of CHO-S HS and heparin showed that HS was deficient in *N*-sulfate and *O*-sulfate when compared to heparin (26). Since sulfate levels are largely determined by the expression levels of the modification enzymes, these data indicate that overexpression of Ndsts, Hs6sts, and Hs3sts may convert CHO cell HS into a heparin-like product.

Hs3st1 is the 3-*O*-sulfotransferase isozyme responsible for producing anticoagulant heparin (5). Previous attempts at engineering CHO-S cells to produce recombinant heparin were partially successful (26). These studies compared the expression of heparin/HS biosynthetic ezymes in rat mast cells to CHO-S cells by RT-PCR. *Hs3st1* was highly expressed in rat mast cells but undetectable in CHO cells. Curiously, Hs3st1 epitopes were detected by Western blotting but this most likely represents inactive enzyme as *Hs3st1* enzyme activity is undetectable in CHO-S and AT3 binding is near zero. Ndst2 was also highly expressed in rat mast cells but undetectable in CHO-S by RT-PCR and by Western blotting. CHO-S cells were transfected to overexpress enzymes Ndst2 and Hs3st1. Two double transfected cell lines were characterized, where both showed significantly increased *N*-sulfation, AT3 binding and Factor Xa inhibition relative to the host CHO-S cell line. However, AT3 binding and Factor Xa inhibition were still well below USP standards for heparin, presumably due to the lack of 6-*O*-sulfation, which decreased in the transfected cell lines. 3-*O*-sulfate levels could not be analyzed because 3-*O*-sulfate inhibits the heparin lyases that digest heparin/HS to disaccharides. On the other hand, *N*-sulfation was dramatically increased; potentially reaching levels that inhibited subsequent *O*-sulfation. Although regulation of the heparin/HS biosynthetic pathway is not completely understood, a leading hypothesis is that Ndst is involved in the termination of sulfation as well as the initiation of sulfation (2). The partial increase in AT3 binding and Factor Xa inhibition and the corresponding decrease in 6-*O*-sulfation observed in the double transfectants along with the known importance of 6-*O*-sulfation in the AT3 binding pentasaccharide (see above) suggests that increasing 6-*O*-sulfation by over expression of Hs6st1, Hs6st2, Hs6st3, or combinations may be required to achieve the USP standards for heparin. This study also shows that it will be important to achieve a balance of expression levels between transgenes and endogenous genes. In *E. coli* and *Saccharomyces cerevisiae*, the expression levels of enzymes involved in metabolic pathways have been controlled by gene titration, promoter engineering, or transcriptional regulation (27–29).

Introducing recombinant heparin to compete with inexpensive unfractionated heparin could be difficult but engineering heparin with fewer side effects could justify a somewhat higher cost making it more competitive. HIT is a significant side effect of heparin. In HIT, the blood platelet count drops below the normal range 5–14 days after the first heparin administration. This can lead to blood clots, stroke, heart attack, deep vein thrombosis and pulmonary emboli, leading to loss of life and limb (30). Treatment requires alternative anticoagulants. Overall, the absolute risk of HIT is 2–3% for unfractionated heparin however, this is a significant risk since hospitals use unfractionated heparin almost exclusively for applications from cardiac surgery to hemodialysis to coating IV tubing. Treating HIT costs around $40,000 per patient. Testing the susceptible patients (10–15%) adds to the costs.

HIT is an immune response against heparin when it is complexed with platelet PF4 (31). Low molecular weight heparin (LMWH) is thought to have reduced risks of eliciting HIT (0.2–0.6%) (32, 33); however, not all researchers are convinced that this has been adequately demonstrated in the clinic (30). In addition, LMWH is not fully reversible with protamine, leaving some high risk patients at risk of bleeding and limiting the use of LMWHs in cardiopulmonary bypass surgery (34). We and other researchers have shown that removal of 2-*O*-sulfate from HS results in significant increases in the IC_50_ for PF-4 binding to heparin in competition formats [(35) and TEGA unpublished data]. It has also been shown that 2-*O*-sulfation is dispensable for activation of AT3 in CHO cells transduced to express Hs3st1 (23). Thus, removal of 2-*O*-sulfation should result in low PF4 affinity but AT3 binding should not be affected.

Another strategy for producing a “premium heparin” to compete with inexpensive unfractionated heparin would be to engineer a LMWH or medium molecular weight heparin directly from cells. As discussed above, LMWH may have a 10-fold decreased risk of HIT but is not fully reversible with protamine, both of these properties, attributed to the lower molecular weight (average 2,000–8,000 Da or dp 4–16) (34). One approach would be to engineer recombinant heparin with an intermediate molecular weight—low enough to inhibit PF4 binding (dp < 42) (22) but large enough to permit complete reversal with protamine (dp > 14) (34, 36). It is known that a 50% reduction in the HS biosynthetic enzyme Ext1 reduces HS chain length (37–39), so *Ext1* heterozygous knockout cell lines could be tested. An alternative approach for directly producing LMWH would be to incorporate heparin degrading enzymes in the CHO cell heparin production process. Currently commercial preparation of LMWHs from unfractionated heparin includes various forms of controlled chemical depolymerization. These methods can result in process artifacts due to the harsh reaction conditions (40–42) relative to the milder enzymatic depolymerization conditions (40). Heparin lyase I, isolated from Flavobacterium heparinum is most commonly used (43, 44) although a number of other heparin lyases are available for depolymerizing heparin and HS (45). To overcome the lack of stability of microbally produced heparinase and to reduce costs associated with its industrial use, heparinases have been immobilized (45). Bioreactors containing immobilized heparinase have been developed for deheparinizing blood and for heparin depolymerization for producing LMWHs (46–50). Alternatively, CHO cells could be engineered to produce heparinase and secrete it into the culture medium. Currently CHO cells secrete mammalian heparanase which has variable effects on HS chain length depending on HS composition (51). Lyase digestion would have to be carefully controlled as, for example, heparinase I cleaves the AT3 binding site within the heparin molecule (52, 53). This may suggest using a different heparin lyase or combination of lyases, or alternatively the activity of heparin lyase I may be improved by directed molecular evolution (54).

The second major challenge to produce a commercially viable product is achieving production levels that can compete economically with the current production of animal derived heparin. Hundreds of millions of slaughtered pigs in China translates into low priced heparin. Industry production methods are closely guarded secrets so determining the cost of heparin production is difficult. Online prices for heparin through Pharmacy Checker.com and GoodRx show retail prices ranging from $0.023–$5.50/international unit (IU) for quantities between 10,000 and 60,000 IU. Costs for recombinant heparin API production come primarily from initial equipment outlay operation and maintenance, cell culture media, and supplies and purification and processing. Increasing production efficiency (or decreasing costs) will be critical. Not considering overhead and sales & marketing, we estimate that recombinant heparin production becomes competitive at production levels of 1 g/L.

As HS is produced as a proteoglycan, one way to increase production may be to overexpress the core protein. Mast cells produce heparin on the core protein serglycin, which is not normally made in CHO cells (55, 56). In addition, serglycin from different species contains different numbers of polysaccharide attachment sites. Over expression of early enzymes in the HS biosynthetic pathway, such as the enzymes catalyzing linker addition (see Figure 2), may increase production if one or more is limiting. Another way to potentially increase productivity is to eliminate the production of inessential cell products genetically, that may compete for precursor molecules or otherwise limit HS production. Commercial production will also require substantial scale-up. Currently TEGA is producing HS from CHO cells in suspension, grown in shaker flasks in serum free medium. Studies with CHO cells demonstrate that bioprocess optimization in bioreactors can substantially improve GAG production (57). Currently available media have been optimized for protein production. Recombinant protein production has greatly increased through bioprocess optimization. Polysaccharide production is likely to have different requirements for precursor molecules, nutrients, and growth conditions so developing medias tailored to polysaccharide production is likely to have a large impact.

## Applications For Non- Anticoagulant Recombinant HS

As discussed, non-anticoagulant physiological properties of heparin and HS have been identified retrospectively from patients treated with heparin to prevent blood clotting (58). These properties are active areas of research and clinical testing. Modifying heparin for non-anticoagulant applications is often aimed at reducing or eliminating anticoagulant properties because in these settings, the anticoagulant activity is an unwanted side effect. Engineering HS in cells has advantages because HS can provide similar benefits without affecting blood clotting. In addition to the lack of anticoagulant activity, different compositions of HS may be more or less effective in different therapeutic applications. This is reflected in the fact that HS compositions differ among different tissues and change during development and aging as well as in disease and in response to injury (22, 59).

Relating HS structure to function is complex because carbohydrate chemistry makes sequencing difficult and HS is a heterogenous mixture of polysaccharide chains. Currently HS is characterized by its composition of sulfated disaccharides despite the fact that the binding sites are families of higher order oligosaccharides that are not revealed by the compositional analysis. HS function is determined by the protein binding sites in localized regions along the chains, but the long polysaccharide chains may contain multiple protein binding sites some of which may stimulate while others inhibit particular cell signaling pathways. This complexity is compounded by the heterogeneity, presumably enabling HS from a particular tissue to be involved in multiple cell signaling pathways. None-the-less HS participates in defined physiologically relevant processes which appear to be regulated by the expression and activity of the enzymes in the metabolic pathways. From an applications standpoint, using HS therapeutically may depend on producing HS with a composition tailored to the relevant HS binding proteins or put another way with the right combination of protein binding sites. That composition reflects a particular combination of binding sites may be reflected by the fact that tissue specific compositions are conserved among different species.

HS acts by binding proteins in the extracellular matrix (ECM) and growth factors and cytokines at the cell surface. In the ECM, it may provide a growth factor sink, which could be released by digestion with heparanase or the activities of other heparin/HS modification enzymes such as the SULFs which reduce subsets of 6-*O*-sulfation. At the cell surface, HS typically functions as a coreceptor modifying growth factor affinity/activity albeit more subtly than the growth factor receptor peptides; much like a rheostat (59). Binding interactions between proteins and HS are complex as there is considerable structural variability in the sites on HS that bind different proteins (22). In some cases, binding requires very specific structural modifications, like the binding sites for AT3 and fibroblast growth factor 2 (FGF2) (60, 61). Other proteins have less-specific interactions with domains along the HS polysaccharide. HS is arranged in alternating highly sulfated NS domains, poorly sulfated NAc domains and domains that are a mixture NS/NAc (2). As varied as the binding sites, are the effects HS binding can have on the HS binding proteins. HS binding can increase the half-life of bound proteins, facilitate oligomerization, and act as an allosteric regulator to change the conformation of a bound protein thereby regulating its activity, for example the AT3 inhibitory activity on Factor Xa (62, 63). By immobilizing or tethering proteins, HS can concentrate them spatially, sequester them as a sink, or arrange them to set up gradients such as VEGF to guide angiogenesis (64).

Through research and clinical observations, a number of potential non-anticoagulant clinical applications of heparin/HS have come to light. In acute inflammatory reactions, heparin has been shown to protect against ischemia-reperfusion injury in animal models (65, 66). Heparin has been used in a number of clinical trials and is currently being tested clinically for treating inflammation associated with sepsis (67). In inflammatory disease, heparin has shown clinical benefit to patients with asthma (58, 68–74) and inflammatory bowel disease (75–78).

There is also significant evidence for the benefit of heparins in cancer because of the frequent use of heparin in the prophylaxis of venous thromboembolism in cancer patients (58, 79–85). Heparin protects against metastatic infiltration related to inflammatory diapedesis (86, 87) and also against tumor growth and angiogenesis (88). With regard to wound healing, isolated reports suggest that topical or systemically applied heparin is able to promote healing and reduce inflammation in burn patients (89). Mechanistically, heparin-binding epidermal growth factor located near the site of injury could be released as an autocrine mitogen competitively, with soluble heparin (90). Embryonic stem cells lacking HS do not differentiate but differentiation can be induced with heparin (91, 92). Multiple growth factor signaling pathways are involved where HS is a critical component (91).

In these applications, anticoagulation is usually an unwanted side effect. In addition, heparin is highly sulfated and is therefore likely to influence multiple signaling pathways, some of which may be counterproductive. Thus, engineering HS may provide advantages due to the lack of anticoagulant activity and because the composition can potentially be engineered to tailor it to the application. It is thus important to understand the relationship between HS composition and application. Examples discussed below are from research aimed at determining how HS composition affects physiology and pathophysiology, which could lead to therapeutic HS compositions or applications where HS is a drug target.

### Inflammation

How HS composition relates to physiological function has been studied by altering the expression of enzymes in the HS biosynthetic pathway. Targeted Ndst1 deletion in myeloid cells showed the HS proteoglycans (HSPGs) are important regulators of macrophage stimulation by Type 1 interferon (IFN) (93). In the knockout, a 10–15% decrease in *N*-sulfation, 6-*O*-sulfation and 2-*O*-sulfation was associated with the macrophages in a perpetual activated state, with increased proinflammatory cytokine secretion, enhanced infiltration and foam cell conversion leading to atherosclerotic lesion formation and increased diet-induced obesity. Macrophages constitutively express low amounts of Type I IFNs. Perpetual macrophage activation in the knockout suggests that the capacity of HS to bind Type I IFNs is a mechanism to retain the interferons near their site of secretion preventing them from interacting with their receptors IFNAR1 and IFNAR2. In this model, under resting conditions, highly sulfated macrophage HS maintains Type I IFN in a quiescent state through sequestration of IFN-β. This model is supported by observations that HS sulfation is decreased in advanced atherosclerotic lesions (94, 95) and a correlation between heparanase expression and the expression of inflammatory markers (96). A similar sulfation dependent model may explain HS regulation of Wnt and BMP signaling (97, 98). Wnt signaling through frizzled receptors is inhibited by HS (similarly BMP signaling through noggin receptors is inhibited by HS). A soluble pool of Wnt or BMP is generated by activation of extracellular sulfatases (SULFs) that remove a subset of 6-*O*-sulfate groups in highly sulfated domains. It is not known whether the SULFs are involved in the regulation of macrophage stimulation. Thus, HS is an important regulator of IFN stimulation. Understanding how HS composition is involved in this regulation could suggest new therapeutic avenues for treating atherosclerosis, either by targeting specific enzymes in the heparin/HS biosynthetic pathway or developing highly sulfated heparanoids that bind type I INFs.

In addition to regulating cytokine secretion, heparin is known to reduce inflammation through its ability to bind inflammatory mediators and inhibit various inflammatory cells including neutrophils. LMWH was tested in COPD patients, an airway inflammatory disease predominated by neutrophils, and found to add significant clinical benefit as an add-on therapy to patients receiving standard treatments (70). Heparin is also effective in stopping the recruitment of inflammatory cell types into tissues through binding adhesion molecules involved in interactions between leukocytes and vascular endothelial cells. Heparin binds a number of adhesion molecules including L-selectin (99), a C-type lectin that uses HS as a ligand for leukocyte rolling during the early interactions with the endothelium and binds P-selectin on endothelial cells (100) which mediates leukocyte/platelet interactions. Chemically modified heparin/HS with defined structures were used to help define HS structures involved in these interactions. Animal studies using modified heparin derivatives showed that heparin inhibits inflammation by blocking P and L-selectin interactions and that inhibition critically depends on 6-*O*-sulfation (101).

In cystic fibrosis (CF), high levels of neutrophil elastase (NE) in lung and sputum promote inflammation by up-regulating neutrophilic cytokines, help disrupt the protease-antiprotease balance by activating other proteases and degrading antiproteases as well as elicit a series of additional pathological effects that make NE an established biomarker of CF progression (102–105). Serine proteases are currently an untreated major cause of airway injury in CF, so they may provide drug targets for innovative therapies. A recent study has shown effective inhibition of NE with 2-*O*,3-*O*-desulfated heparin in CF patient sputum but only in combination with the CF treatment dornase (DNAase-I) because DNA and heparin compete for binding sites on NE (106). Polymer length is also important as effective *O*-desulfated heparin chains should be in the range of 15 saccharides.

### Metastasis

Heparin may also be used to target metastasis (107). Selectin dependent interactions are important for metastatic tumor cell extravasation (88). Tumor cells express mucins on their surface which interact with selectins on the surfaces of endothelial cells, platelets and leukocytes. Clinically, mucins correlate with poor prognosis and increased metastasis (108–110). Heparin and heparin derivatives block P-selectin, which prevents interactions with endothelial cells that arrest circulating tumor cells and prevents binding to platelets that form a protective cloak around tumor cells. Again, 6-*O*-sulfation is critical for inhibition of the selectin interactions (101, 111).

### Tumor Angiogenesis

Targeted deletion of Ndst1 in endothelial cells reduced the *N*-sulfate content of cellular HS and reduced tumor growth and tumor angiogenesis without affecting normal angiogenesis (wound healing) (112). Reduction of *N*-sulfation reduced FGF2 and VEGF_164_ binding to cultured endothelial cells that resulted in decreased Erk1/2 phosphorylation and decreased branching and increased apoptosis in branching assays (112). Although non-tumor endothelial cells showed these alterations, physiological angiogenesis remained normal suggesting that tumor dependent factors contributed to the difference; such as a higher demand on tumor vasculature or different compositions of growth factors. Thus, physiological and pathophysiological contributions of HS to cell signaling are complex, depending on HS composition and tissue environments but may none-the-less be exploited. With respect to tumor angiogenesis, targeting HS may have advantages over targeting single growth factors because HS contributes to the activity of numerous proangiogenic factors including FGF2, VEGF, hepatocyte growth factor (HGF), platelet-derived growth factor (PDGF), heparin-binding epidermal growth factor (HB-EGF), angiopoietin, tumor necrosis factor-α (TNF-α), interleukin-8 (IL-8), and others (113). In light of the tumor specificity observed here, inhibitors of particular biosynthetic enzymes in the heparin/HS biosynthetic pathway or antibodies to HS may be more effective than the current anti-VEGF antibodies [Avastin® (bevacizumab), Roche].

### Cancer Stem-Like Cells

A separate study identified a hexasaccharide that selectively inhibits cancer-stem cells (CSCs) (114). Like the study above, Erk1/2 activity was inhibited but the hexasaccharide also produced sustained activation of p38 MAPK. With an understanding that proteoglycans and GAGs modulate important aspects of cancer progression, a screening technology was used, that was developed by the group (115, 116), that enriches for CSCs, because CSCs constitute a small fraction of the tumor cell population. The screening technology identifies agents that specifically target CSCs and not progenitor cells or other cells in the cancer cell population. HS06 contained the repeated disaccharide IdoA2S-GlcNS6S, and demonstrated differential, isoform specific modulation of MAPK family members; increasing p38α and attenuating activation of p38δ, without activating other MAPK family members. The importance of fine structure was clearly demonstrated as similar sized CS and DS oligosaccharides were considerably less effective and longer HS chains displayed reversed activities; as p38 inhibition and ERK1/2 activation.

### Stem Cells

Stem cell maintenance, expansion and differentiation are also areas where heparin and HS are of prime interest both commercially and for research. Embryonic stem cells (ESCs) have been used to characterize the role of HS in regulating differentiation because alterations in HS structure can be lethal in mice (91). ESCs are an ideal alternative as they can self-renew so they can be maintained in an undifferentiated state and they are pluripotent. Mutating enzymes in the HS biosynthetic pathway causes numerous developmental defects and stem cells with these mutations can be derived from the embryos of mutant mice (91). Knocking out HS completely and then adding stimuli that promotes differentiation leaves the cells in a primed state but limits further differentiation. Cells lacking *N*-sulfation also lack 2-*O*-sulfation as 2-*O*-sulfation depends on *N*-sulfation (see above) however the cells maintain half of the normal 6-*O*-sulfation. These cells maintain their ESC characteristics under conditions that maintain pluripotency however angiogenic differentiation is impaired. Thus, sulfated HS is required for normal development. Knocking out 2-*O*-sulfation resulted in an increase in *N*-and 6-*O*-sulfation with proliferative defects and defects under conditions promoting neural differentiation. Under these conditions haemopoietic differentiation appeared normal. Consistent with the increase in 6-*O-*sulfation, neural defects were also observed in SULF enzyme mutants, that also increased 6-*O*-sulfation. However, under these conditions, defects in haemopoietic differentiation were also evident. This may suggest the need for tighter control of 6-*O*-sulfation (91).

As discussed, stem cell differentiation is often promoted by the addition of costly growth factors and heparin, however, adding lower concentrations of growth factors with polysaccharides selected for activation of those growth factors may be more effective and cost effective. For example, mesodermal differentiation in HS deficient cells was restored by the addition of heparin or HS presumably by restoring correct processes involving Wnt, BMP4, and Nodal signaling (117). Using a panel of HS compositions, the requirement for 6-*O*- and *N*-sulfation were identified with HS being more effective than heparin; again indicating that the organization of the sulfate groups on the polysaccharide chains is important, not just high levels of sulfation. Neural differentiation required some distinctly different structures. Addition of heparin/HS to HS-containing cells significantly improved neural differentiation where *N*-, 2-*O*- and 6-*O*-sulfations were all important. Thus, there is a strict requirement to specifically tailor the composition of exogenous HS added, depending on the type of differentiation (91).

Satellite cells (SC) are muscle stem cells that lie quiescent in muscle fibers. In response to injury, SCs are activated, proliferate as myoblasts and form new muscle fibers (118). Understanding how SC activation is regulated is of clinical interest because during aging, skeletal muscle loses regenerative capacity due to SC impairment and altered molecular signaling in muscle (119–122). A number of factors have implicated HS in this process. HS from SC-derived proliferating myoblasts has equal levels of non-sulfated and sulfated disaccharides whereas cultures of differentiated myotubes express a much higher ratio of sulfated disaccharides relative to non-sulfated disaccharides. This was primarily due to increased levels of mono-sulfated disaccharides, that were principally 6-*O*-and 2-*O*-sulfated. 6-*O*-sulfation continues to increase with age which corresponds with the decreasing regenerative capacities of SCs. Further examination revealed evidence that enhanced FGF2 signaling due to increased 6-*O*-sulfation (6-*O*-sufated HS is required for fibroblast growth factor receptor, FGFR, binding and FGF2 signaling) in the aging mice exhausted the quiescent SCs leading to fibrosis and sarcopenia (123).

To identify key HS structures, heparin was chemically modified to form HS mimetics (124). Since heparin is over sulfated HS, the mimetics contained the variant types of HS found in tissues including: heparin, completely desulfated heparin/HS and heparin/HS forms that were *N*-sulfated; 2-*O*-sulfated; 6-*O*-sulfated; *N*- and 2-*O*-sulfated; *N*- and 6-*O*-sulfated; *N*-, 2-*O*- and 6-*O*-sulfated and over-sulfated heparin with additional 3-*O*-sulfation. All of the mimetics except over-sulfated heparin reduced differentiation. In most cases, decreased differentiation was accompanied by increased proliferation. Over sulfated heparin did not reduce differentiation but reduced cell numbers. One mimetic: *N*-acetylated-2-*O*-desulfated (with increased 6-*O*-sulfation like aging) inhibited differentiation and did not promote proliferation. Since myoblast proliferation is strongly promoted by FGF2, over-sulfated heparin was tested to see if its antiproliferative effects were mediated by inhibition of FGF2 signaling. Over-sulfated heparin reduced Erk1/2 phosphorylation presumably by acting as an extracellular sink for FGF2. In contrast, *N*-acetylated heparin which inhibits differentiation, prolonged Erk1/2 phosphorylation consistent with promoting cell expansion. These results strongly suggest that myoblast proliferation is regulated by HS though FGF2 signaling. The HS mimetics were then tested for their ability to promote or inhibit SC self-renewal since aging is accompanied by reductions in SCs. In cell culture, self-renewal consists of myoblasts that exit the cell cycle but instead of differentiating they enter a quiescent state. Quiescence can be detected by the expression of transcription factor Pax7 (125). Although most of the HS mimetics inhibited differentiation the results on self-renewal varied. 2-*O*-sulfated derivatives inhibited self-renewal while *N*-sulfated derivatives promoted it. Thus, HS has multiple roles in cell differentiation and cell fate determination which critically depends on composition. These results suggest that *N*-sulfated HS or heparanoids could promote self-renewal of SC. Alternatively, inhibitors of 2-*O*-sulfation may also be effective.

Different growth factor binding affinities were identified in differentially regulated neural precursor cell HS from E9 and E11 mouse neuroepithelium (126). These affinities correlate with growth factor expression where FGF2 expression begins at E9 and continues throughout development while FGF1 expression begins at E11 when neurons begin to differentiate. HS expression appears to match growth factor expression as HS prepared from cultured E9 neuroepithelial cells bound FGF2 four times more strongly than FGF1 whereas HS from E11 cells bound FGF1 six times more strongly than FGF2. Correspondingly, E9 HS was seven times more effective at stimulating cell proliferation with FGF2 consistent with the observation that FGF2 regulates neural precursor cell division (127–129). This E9 HS was subsequently used to preferentially expand human mesenchymal stem cells (hMSCs) (130), which, unlike conventional methods with a cocktail of protein factors that typically result in heterogeneous cultures, were particularly effective in a bone repair model.

### Bone Wound Healing

HS has also been tested with clinically applied growth factors. In the US, a significant percentage of bone fractures show healing deficiencies (131). Standard-of-care calls for orthopedic rod or plate supported allograft or autograft bone (132), however, complications limit these methods (133–135). Subsequently, growth factor-based treatments have been tested for promoting bone formation. Endogenous bone morphogenic protein (BMP) levels can be limiting at bone trauma sites and BMP is inhibited by secreted antagonists like noggin and gremlin (136, 137). Devices containing BMP-2 are FDA approved, however rapid degradation and poor pharmacokinetics limit their effectiveness so there is concern that excessive dosing used to compensate for these limitations can lead to adverse events (138, 139). In addition, using high levels of growth factors is expensive (140). Heparin has been used to promote BMP-2 efficacy. BMP-2 is a heparin binding protein and heparin improves its bioavailability by blocking BMP-2 binding to cell surface HS, stabilizes and protects the growth factor and reduces inhibition by the antagonist noggin. These are general effects that heparin has on heparin binding growth factors which have led to heparin containing biomaterials aimed at improving BMP-2 efficacy. BMP-2 mediated bone formation is enhanced by heparin however, heparin's osteoporotic and anticoagulant activities along with side effects due to a wide range of heparin binding proteins has limited its clinical use in bone regeneration. BMP-2 also binds HS, implicated by the fact that HS is tissue specific and developmentally regulated in stem cells and osteoprogenitor cultures (141–145). Temporal coordination of HS expression with biomarkers of bone healing is consistent with selective HS growth factor interactions. These interactions stabilize BMP-2 and prolong its half-life while recruiting the BMP receptor to enhance downstream signaling. HS also has less side effects than heparin but preparations from commercial sources would be less efficacious due to the extensive structural heterogeneity among the HS chains (144). To obtain a more homogeneous preparation with a stronger affinity for BMP-2, peptides derived from the BMP-2 protein sequence were used to selectively enrich porcine mucosal HS for a BMP-2 binding fraction. The bound fraction exhibited many of the intended properties including a major enhancement of bone repair and so this method may constitute a platform technology in which peptides derived from heparin binding proteins are used to enrich HS for high affinity fractions. Disaccharide analyses identified compositional differences between the original material, the unbound fraction and the bound fraction with the major difference being a significant increase in a trisulfated disaccharide (ΔHexUA, 2S-Glc-NS, 6S) in the bound fraction.

## Conclusions

A number of challenges remain for developing HS based therapeutics. Relating composition to non-anticoagulant heparin/HS function is an ongoing process and the subtler structural aspects are still not very well-defined. For pharmaceutical heparin, approximately one AT binding site in a third of the chains is sufficient for anticoagulant effects. The challenge with recombinant heparin is increasing the level of sulfation of HS from mammalian cells to achieve that frequency. With HS, progress has been made using HS binding proteins to identify binding sites and select HS fractions. Binding may be only part of the story however as growth factor binding can have different effects in different cellular contexts. Heparin/HSs are known to bind a range of growth factors, but this does not always result in signaling. For example, HS functions as a coreceptor for FGF signaling by interacting with FGF2 and FGFR1 to form cell surface ternary complexes. HS binding to FGF2 requires *N*- and 2-*O*-sulfation whereas signaling requires simultaneous FGFR binding which requires 6-*O*-sulfation (146, 147). In addition, available commercial HS is currently a byproduct of heparin production from animal tissue and of poor quality.

Recently, studies using libraries of engineered cell lines have been reported that could be used to help determine HS fine structure involved binding protein ligands. By knocking out genes encoding particular biosynthetic enzymes in the HS and CS/DS biosynthetic pathways and then introducing others (28 genes individually and in select combinations) the GAGome library of engineered CHO cells was used to display complex GAG features and determine which features are relevant for specific binding interactions and the biosynthetic enzymes responsible for their production (148). Similarly, gene knockout of all of the HS biosynthetic enzyme isoforms individually and in combinations was used to generate a library of HS mutant mouse endothelial cell lines. This library comprises cells that produce a diverse array of HS compositions which again have been used to determine which specific modifications or HS fine structure as well as overall sulfation levels are relevant to interactions with protein ligands (149). Unlike oligosaccharide arrays, these cell libraries display native sized HS chains with multiple binding sites which may better correlate composition with physiological function and thus may become increasingly important resources.

A number of strategies for producing recombinant heparin have been reported. In an attempt to increase production, HEK293 and HEK293T cells were transfected with human serglycin; His-tagged to facilitate analyses of the attached GAG chains (55). Eighty-five percent of the serglycin was secreted as proteoglycan with the attached chains comprised of 50% HS chains and 50% CS/DS. Trisulfated disaccharides were observed however there was a tendency toward less sulfated disaccharides relative to the LMWH, Deligoparin, and the anticoagulant potency was 7-fold lower by weight than unfractionated porcine heparin as determined in a fibrin clot assay. HEK293 cell lines are used for production however these results indicate that additional engineering would be required.

Bacteria produce polysaccharides, which however, tend to be structurally simple and non-sulfated such as heparosan, hyaluronan and chondroitin, as prokaryotes lack the machinery found in Golgi responsible for sequential sulfation (150). Thus, metabolic engineering of prokaryotes would not only require the introduction of new enzymes but would also require a strategy for compartmentalizing the enzymes to achieve a sequential synthetic pathway. On-the-other-hand, chemoenzymatic methods are being tested to produce heparin in the laboratory from heparosan, the capsular polysaccharide of *E. coli* K5 (151–154). Heparosan is first chemically converted to N-sulfoheparosan and then is modified in a three step enzymatic process to obtain anticoagulant heparin (155). Small quantities of material, closely resembling animal sourced heparin have been demonstrated (156) but scale up remains a major challenge. Multistep enzymatic synthesis is already an expensive proposition for manufacturing and in addition, substantial quantities of *E. coli* expressed enzymes are required. Initial attempts to scale up to *E. coli* production of two critical enzymes in fed-batch stirred tank fermenters was unsuccessful, resulting in low levels or inactive enzymes (155).

More recently chemoenzymatic synthesis of short oligosaccharides have shown promise. The dodecasaccharide (12-mer) 12-mer-1 displays anticoagulant activity similar to LMWHs, is protamine-reversible and is amenable to multigram-scale chemoenzymatic synthesis (157). Scale up is still a challenge as this is a 22-step process and requires the separate expression of the heparin biosynthetic enzymes. As the authors confirm, industrial scale synthesis would require substantial optimization of the enzyme expression and purification methods, but perhaps there is precedence for this in fondaparinux which is currently produced at the kilogram scale (158). Fondaparinux production involves numerous steps and initial laboratory synthesis was at the milligram scale (159).

TEGA's strategy is to engineer CHO cells to produce recombinant heparin/HS, which enables the entire supply chain to be under GMP control. With cell sourced HS, we achieve a high level of structural consistency in size and composition of the polysaccharide chains. Like the other strategies for producing recombinant heparin/HS, achieving sufficiently high and cost-effective production levels will be a challenge. Some of this may involve bioprocess development and scale-up along with cell engineering. We hope to accelerate the cell engineering process by introduction of multiple genes simultaneously and then using high throughput screening and analysis methods we have developed.

## Author Contributions

The author confirms being the sole contributor of this work and has approved it for publication.

### Conflict of Interest Statement

CG was employed by company TEGA Therapeutics, Inc. Work reported in this article is covered under patent applications: 1. Patent applications: PCT/US2016/067373 Cellular Glycosaminoglycan Compositions and Methods of Making and Using 12/16/2016 TEGA Therapeutics, Inc. 2. PCT/US2017/066860 *in vitro* Heparin and Heparan Sulfate Compositions and Methods of Making and Using 12/15/17 TEGA Therapeutics, Inc.
